# Efficacy of two topical fluralaner formulations (Bravecto®; Bravecto® Plus) against Asian longhorned tick (*Haemaphysalis longicornis*) infestations of cats

**DOI:** 10.1186/s13071-023-05658-8

**Published:** 2023-01-26

**Authors:** Melissa Petersen, Riaan Maree, Henda Pretorius, Julian E. Liebenberg, Frank Guerino

**Affiliations:** 1 Merck Animal Health, De Soto, KS 66018 USA; 2 Clinvet USA, Waverly, NY 14892 USA; 3 Clinvet South Africa, Bloemfontein, 9338 South Africa; 4 Merck Animal Health, Madison, NJ 07940 USA

**Keywords:** Asian longhorned tick, Bravecto®, Cat, Efficacy, Fluralaner-moxidectin, Fluralaner topical, *Haemaphysalis longicornis*

## Abstract

**Background:**

The invasive tick species, *Haemaphysalis longicornis*, is becoming established in the USA, presenting a growing threat to dogs and cats. Two 90-day studies were initiated, the same protocol in each, to confirm the efficacy of a single application of two fluralaner formulations against *H. longicornis* infestations of cats.

**Methods:**

Cats were randomized among three groups in a 1:1:1 ratio (10 cats/group). Group 1 cats were untreated controls; Group 2 cats were treated with a topical fluralaner formulation (Bravecto®); Group 3 cats received a topical formulation containing fluralaner and moxidectin (Bravecto® Plus). Treatments were administered once (Day 0) at the label dose rates. Each cat was infested with 50 *H. longicornis* ticks on Day 7 for study qualification and also infested with 50 ticks on Days 2, 28, 58 and 88. Tick counts were completed on Days 5, 2, 30, 60 and 90. The primary objective was based on percentage reductions in arithmetic mean tick counts.

**Results:**

Pre-study infestations showed all study cats were susceptible to tick challenge. Except for Day 2 in one study, at least six control cats retained ≥ 25% of each challenge, demonstrating an adequate infestation for efficacy assessments. Across studies on Days 2, 30, 60 and 90, the mean live tick infestation rate (number of ticks recovered from each cat/infesting challenge to each cat) of Group 1 cats ranged from 25.0 to 69.6%. Efficacy of each formulation, based on live tick counts, was 100% on Day 2 and > 95 to 100% at each subsequent assessment. Between-group differences were statistically significant (*P* < 0.0001) for each treatment versus control comparison.

**Conclusion:**

At the label dose rate, both topical fluralaner formulations were 100% effective in eliminating *H. longicornis* ticks from cats infested at the time of treatment. Efficacy of > 95 to 100% was then maintained through 90 days following a single application. Fluralaner is therefore a treatment of choice for protecting cats against this invasive tick species.

**Graphical Abstract:**

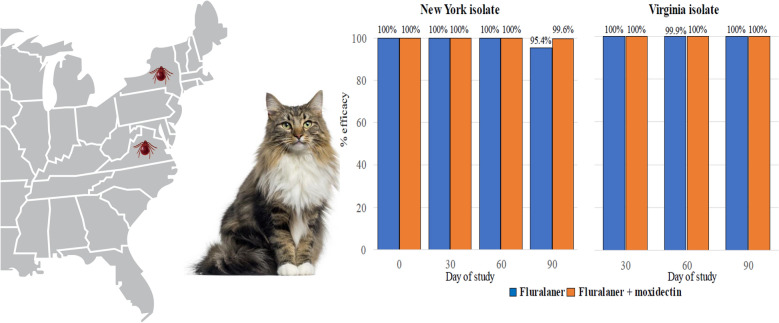

## Background

*Haemaphysalis longicornis*, known as the East Asian or longhorned tick, is native to Japan, China, eastern Russia and Korea but has invaded and established in other parts of the world (i.e., Australia, New Zealand and other Pacific islands) [1, 2]. This invasive tick species was first confirmed in the US in 2017 on a sheep on a New Jersey farm and has since been identified in 17 states on multiple species of animals including dogs, cats and humans [1, 3, 4]. A vector for tick-borne pathogens such as *Theileria orientalis* and *Rickettsia rickettsii*, *H. longicornis* has the potential for rapid population increases as it can reproduce through parthenogenesis [4]. Additionally, there is concern that its presence will expand quickly to different geographical locations because numerous wildlife species, including birds, can be infested and spread the tick to new locations [4]. At the time of the study there was no product in the USA labelled for efficacy against infestations with this tick species.

Fluralaner is an isoxazoline ectoparasiticide that was first approved for use in the USA in 2014 as an orally administered chewable tablet for dogs, with a topical formulation for dogs and cats subsequently approved in 2016. Both formulations have been shown to provide extended activity of up to 12 weeks acaricidal and insecticidal activity [5, 6]. A more recent addition to the fluralaner product portfolio is a formulation containing a combination of fluralaner and moxidectin. This combination product is now approved for use in cats and kittens in several countries around the world. In addition to prevention of heartworm disease and anthelmintic activity against existing infections, a single administration is labeled to provide insecticidal and acaricidal activity for 2 months in the USA and 12 weeks in Europe, Australia and other countries, based on the specific indications and regulatory requirements in those countries [7, 8]. While its acaricidal activity is already well established, there have been no reports demonstrating that fluralaner or any other isoxazoline is effective against feline *H. longicornis* infestations. As this tick has been found on cats in the USA, a study was initiated to determine and demonstrate the efficacy of each topical fluralaner formulation against two different US isolates of *H. longicornis* infestations present at the time of treatment and against challenges for 3 months following a single treatment.

## Methods

The objective of both studies was to evaluate the effectiveness of topical solutions of fluralaner as a single entity product and fluralaner in combination with moxidectin in cats infested with *H. longicornis* ticks. Each study was randomized with an untreated control group. All observations and procedures, including general health observations, tick infestations and tick counts, were performed by masked individuals. The same protocol was used in each study, both of which were conducted in accordance with Good Clinical Practices and with the guidelines of the World Association for the Advancement of Veterinary Parasitology (WAAVP) for evaluating the efficacy of parasiticides against flea and tick infestations of dogs and cats [9, 10].

### Animals

On or before Day -8 in each study, 36 Domestic shorthair cats were bathed in a non-medicated shampoo and moved to cages for an acclimation period. The floor size of each cage was approximately 1.1 m × 1.2 m and at least 2.0 m high. To be included in either study cats had to be clinically healthy with no pre-existing conditions (e.g. injury, trauma, disease) that could have affected the study, at least 6 m of age, not treated with any long-acting anti-flea or anti-tick product within the previous 120 days and have demonstrated susceptibility to tick infestation based on retaining at least 13 live ticks (at least 25% of a pre-study infestation) placed on Day -7. Cats were excluded if showing evidence of any concurrent disease, including skin conditions such as lesions or poor hair coat, if lactating or pregnant, or if fractious to a point that behavior could affect handling for study procedures. From placement for acclimation and throughout the study, cats were housed in individual cages in a thermostatically controlled environment with an approximate 12-h light/12-h dark cycle, fed a commercial cat food per site practice and allowed ad libitum access to water. Cages were segregated by treatment group to avoid cross exposure of cats to fluralaner. From those presented for acclimation, 30 cats in each study were retained for experimental purposes. Cats selected for Study 1 were 8 males and 22 females, aged from 17.0 to 41.2 months (median age in each group was approximately 28 months) and weighing from 2.5 to 9.1 kg (median weights in each group from 3.5 to 3.8 kg) on Day 2. Study 2 cats were 7 males and 23 females, aged from 12 to 84 months (median age in each group was approximately 28 months) and weighing from 2.3 to 4.5 kg (median weights in each group from 2.9 to 3.2 kg).

### Randomization and treatment

On Day -5 or -4, 30 cats that satisfied the inclusion criteria were selected for each study. On Day -5 or -3 cats were randomized, using a computer-generated randomization table, to treatment groups in a 1:1:1 ratio, so that 10 cats were included in each group. Cats in Group 1 remained untreated but were handled in the same manner as cats in groups allocated to one of the fluralaner-treated groups. Group 2 cats received a single application of a topical formulation of fluralaner, 280 mg/ml, at the minimum label dose of 40 mg/kg (Bravecto topical solution for cats; Merck Animal Health, Madison, NJ, USA). Cats randomized to Group 3 were treated at the minimum label dose with a single application of the topical combination product of fluralaner (280 mg/ml; dose rate 40 mg/kg) and moxidectin (14 mg/ml; dose rate 2.0 mg/kg) (Bravecto Plus for cats, Merck Animal Health, Madison, NJ, USA). With doses calculated from the Day -2 body weights, treatments were applied on Day 0, by parting the hair and placing the tip of a disposable syringe at the base of the skull and squeezing the plunger to apply the contents, taking care to avoid any run-off. Administered fluralaner dose rates in Group 2 cats ranged from 39.6 to 40.3 mg/kg and in Group 3 cats from 39.8 to 40.4 mg/kg. Each cat (all treatment groups) was kept on the treatment table for approximately 3 min following treatment administration before being returned to its cage. Any abnormal observations such as solution drip-off or shake-off and cat behavioral changes were recorded. Any abnormal observations were recorded as an adverse event, and any run-off or product loss through head shaking was recorded, but no further treatment was applied to replace the estimated loss of dose.

Immediately after the treatment procedure cats were returned to their cages and observed for approximately 1 h for adverse events and to ensure the treatment was retained. Each cat was observed for general health by masked personnel on the day of treatment administration at 1 h (± 15 m), 3 h (± 30 m) and 6 h (± 30 m) post-treatment and then daily through the end of the study. Treatment sites of all cats were also examined on Days 1, 2, 3, 7 and 14 post-treatment. Post-treatment observations on all cats were conducted in a random order of evaluation.

### Tick infestations and counts

At the pre-treatment infestation on Days -7, and on Days -2, 28, 58 and 88, each of the 30 cats included in each study was infested with approximately 50 adult, unfed *H. longicornis* ticks. The *H. longicornis* isolates in Study 1 had been collected in New York during January 2020 and those in Study 2 from a colony originally collected from vegetation in Virginia, USA, in October 2018. Prior to placing the infestation, each cat was sedated (dexmedetomidine hydrochloride, 0.12 ml/kg Dexdomitor®, Zoetis), and a vial containing the ticks was emptied onto its back. After placing the ticks, an Elizabethan collar was fitted to each cat to prevent self-grooming and to allow the ticks to find attachment sites, and the cat was then moved into an individual infestation chamber for a period of up to 4 h to ensure that ticks could establish. The collars remained in place until the tick counts for each infestation were completed. The exception was the Day -2 infestation when collars were removed prior to treatment on Day 0 to avoid interference with the treatment retention. Collars remained off the cats for the subsequent 48 h prior to the Day 2 tick counts. Before each use, infestation chambers were thoroughly washed with soap and water, rinsed with clean water and double rinsed with isopropyl alcohol (with air drying between each alcohol rinse), and collars were thoroughly washed with soap and water, rinsed with clean water and doubled rinsed with isopropyl alcohol (with air drying between each alcohol rinse). Each collar and each infestation cage were used for the same cat for the duration of the study.

Tick counts were completed on Day 2 and on Days 30, 60 and 90 (at 48 ± 4 h post-infestation). For each cat, after the head, dorsal and dorso-lateral aspects had been visually examined, the cat was turned onto its back for ventral and ventro-lateral examination. The cat was then thoroughly combed using a tick comb (at least nine teeth per cm) to remove any ticks that might have been missed during the visual examination. Each examination and combing procedure lasted for at least 5 min. Collected ticks, whether attached or free on the cat, were counted and classified as live or dead.

### Number of cats

According to guidelines issued by the World Association for the Advancement of Veterinary Parasitology (WAAVP), for studies investigating the efficacy of ectoparasiticides, a minimum of six cats is recommended for each treatment group [10]. A sample size of 10 cats per treatment group was used in this study to help ensure sufficient data were available to justify a statistical assessment.

### Statistical analysis

The primary endpoint in each study was based on arithmetic mean live tick counts. For between-group comparisons to be valid, on each tick challenge day an infestation rate of at least 25% of the infesting burden (at least 13 live ticks) was required to be collected from at least six control cats. Live tick counts of each treated group were compared to those of the control group using a linear mixed model that included treatment group as a fixed effect. Testing was two-sided with a 5% level of significance. The null hypothesis was that there was no significant difference between each treated group compared with the control group. Separate analyses for each fluralaner treatment group hypothesis were conducted (fluralaner formulation compared to the control group) at each tick count day. Least square means from the model were used for percent effectiveness calculation. The primary software used for analysis was SAS version 9.4. or higher.

Within each infestation schedule, the primary efficacy endpoint was calculated using the formula below, based on arithmetic mean tick counts:

$${\text{Efficacy }}\left( \% \right)\, = \,\left[ {\left( {{\text{mean Group }}1{\text{ counts}}{-}{\text{mean Group }}2{\text{ counts}}} \right)/{\text{mean Group }}1{\text{ counts}}} \right)]\, \times \,100.$$

## Results and discussion

The dorsal midline of each cat was examined on Days 0, 1, 2, 3, 7 and 14 for evidence of coat or skin changes at the site of product application. No abnormal treatment site observations were noted, and no treatment-related adverse events observed. The initial infestations (Days -8 to -3) demonstrated the susceptibility of all study cats to the laboratory infestations, in Study 1 ranging from 13 to 36 live attached ticks and in Study 2 from 13 to 37 live attached ticks. In Study 2, on Day 2, too few of the Group 1 cats had retained the protocol-mandated requisite number of live ticks to allow treatment efficacy assessments. The decrease in tick counts and infestation rates noted on Day 2 in the Group 1 control cats in both studies was attributed to the removal of the Elizabethan collars on Day 0, which allowed for grooming of the ticks over the final 2 days of the infestation (Day 0 to 2). At all other assessments in both studies, at least 13 live *H. longicornis* ticks were collected from at least six Group 1 cats, thus validating between-group comparisons. On Days 2, 30, 60 and 90, the mean live tick infestation rate (number of ticks recovered from each cat/infesting challenge for each cat) of cats in the negative control group in both Study 1 and 2 ranged from a low of 25.0% to a high of 69.6% (Table 1).

**Table 1:** *Haemaphysalis longicornis* infestation rate in control cats

Day of count	Number (%) of control group cats with adequate infestations		Mean infestation rate (%)^a^	
	Study 1	Study 2	Study 1	Study 2
2	7/9^b^ (77.8)	3/10 (30.0)	34.9	25.0
30	8/10 (80.0)	9/10 (90.0)	65.6	69.6
60	9/10 (90.0)	7/10 (70.0)	47.8	40.8
90	8/10 (80.0)	9/10 (90.0)	47.4	43.2

^a^Infestation rate calculated as: (number of ticks recovered from each cat/infesting challenge for each cat) × 100

^b^Data from one cat were missing on Day 2 only

In both studies, at each post-treatment assessment on which Group 1 *H. longicornis* infestations met the protocol-required numbers, the reductions in live tick counts from both treated groups relative to Group 1 were statistically significant (*P* < 0.0001) (Table 2). Based on arithmetic mean live tick counts, fluralaner efficacy was 100% against existing *H. longicornis* tick infestations at 48 h post-administration, and > 95 to > 100% efficacy was maintained for the full 90-day study period (Table 3).

**Table 2:** Comparison between of least square mean estimates of live *Haemaphysalis longicornis* tick counts (10 cats per treatment group)

Day of counts	Control vs. fluralaner				Control vs. fluralaner + moxidectin			
	Estimate	Standard error	Test statistic	P-value	Estimate	Standard error	Test statistic	*P*-value
Study 1								
2	17.4	2.8	t_17_ = 6.28	< 0.0001	17.4	2.8	t_17_ = 6.28	< 0.0001
30	32.9	5.5	t_18_ = 5.93	< 0.0001	32.9	5.5	t_18_ = 5.93	< 0.0001
60	24.0	3.3	t_18_ = 7.21	< 0.0001	24.0	3.3	t_18_ = 7.21	< 0.0001
90	22.6	3.8	t_18_ = 6.00	< 0.0001	23.6	3.7	t_18_ = 6.38	< 0.0001
Study 2								
2^a^	12.5	3.1			12.5	3.1		
30	34.8	3.6	t_18_ = 9.68	< 0.0001	34.8	3.6	t_18_ = 9.68	< 0.0001
60	20.2	3.3	t_18_ = 6.18	< 0.0001	20.4	3.3	t_18_ = 6.26	< 0.0001
90	21.6	3.0	t_18_ = 7.26	< 0.0001	21.6	3.0	t_18_ = 7.26	< 0.0001

^a^For statistical comparisons, the protocol requirement for each study was that at least six cats in the control group had to be adequately infested with live *H. longicornis* ticks (≥ 13 ticks). At this time point in Study 2, only three control group cats had adequate infestations

**Table 3:** Efficacy of topical fluralaner formulations against *Haemaphysalis longicornis* counts in cats (10 cats per treatment group)

Day of counts	Arithmetic mean live tick counts			% efficacy	
	Control	Fluralaner	Fluralaner + moxidectin	Fluralaner	Fluralaner + moxidectin
Study 1					
2	17.4	0.0	0.0	100	100
30	32.9	0.0	0.0	100	100
60	24.0	0.0	0.0	100	100
90	23.7	1.1	0.1	95.4	99.6
Study 2					
2^a^	12.5	0.0	0.0		
30	34.8	0.0	0.0	100	100
60	20.4	0.2	0.0	99.9	100
90	21.6	0.0	0.0	100	100

^a^For efficacy assessments based on control-group comparisons, the protocol requirement for each study was that at least six cats in the control group had to be adequately infested with live *H. longicornis* ticks (≥ 13). At this time point in Study 2, only three control group cats had adequate infestations

A national USA survey has demonstrated the susceptibility of cats to tick infestations, even in those believed by owners to spend no time outside, and regardless of gender and whether spayed or neutered, and *H. longicornis* has already infested cats in the USA [4, 11]. In Japan where *H. longicornis* is regarded as being a native species, it has become the most common tick that infests cats [12]. The discovery and continuing spread of *H. longicornis* therefore amplifies the need for implementation of feline tick control measures in the USA.

Cat owners in the USA are believed to have a generally poor compliance with flea and tick treatment recommendations and prefer convenience and longer duration of efficacy than is provided by monthly control measures [13]. Thus, the convenience of a topical formulation and the sustained high efficacy over a 3-month post-treatment period, shown by both formulations tested in this study, address a need of cat owners. This high and sustained efficacy can therefore be an asset in controlling tick infestations and reducing the associated risk of exposure to tick-borne pathogens that may be carried by *H. longicornis* and other tick species.

## Conclusion

At the minimum recommended label dose rate of 40 mg/kg, two topical formulations of fluralaner, one a single entity formulation, the other in combination with moxidectin, were 100% effective in eliminating two different US isolates of *H. longicornis* ticks from cats infested at the time of treatment. Efficacy > 95 to 100% was then maintained throughout 90 days following one application. Each formulation therefore provides an effective means of managing the risk of infestation posed by this increasingly common invasive tick species.

## Data Availability

Data from this study are proprietary and maintained by Merck Animal Health, Madison, NJ, USA.
