# Publisher Correction: Efficacy of two topical fluralaner formulations (Bravecto®; Bravecto® Plus) against Asian longhorned tick (*Haemaphysalis longicornis*) infestations of cats

**DOI:** 10.1186/s13071-023-05702-7

**Published:** 2023-02-21

**Authors:** Melissa Petersen, Riaan Maree, Henda Pretorius, Julian E. Liebenberg, Frank Guerino

**Affiliations:** 1 Merck Animal Health, De Soto, KS 66018 USA; 2 Clinvet USA, Waverly, NY 14892 USA; 3 Clinvet South Africa, Bloemfontein, 9338 South Africa; 4 Merck Animal Health, Madison, NJ 07940 USA

**Publisher Correction: Parasites & Vectors (2023) 16:36** 10.1186/s13071-023-05658-8

Following publication of the original article [1], the authors flagged that during production of their article the negative signs, '−' (which indicate days prior to treatment Day [0]), had been erroneously removed from the following sentences of the Methods subsection of the Abstract: “Each cat was infested with 50 H. longicornis ticks on Day −7 for study qualification and also infested with 50 ticks on Days −2, 28, 58 and 88. Tick counts were completed on Days −5, 2, 30, 60 and 90. The primary objective was based on percentage reductions in arithmetic mean tick counts.”

The sentences have since been corrected in the published article. The publisher thanks you for reading this correction and apologizes for any inconvenience caused.
